# Emergence of the Mitochondrial Reticulum from Fission and Fusion Dynamics

**DOI:** 10.1371/journal.pcbi.1002745

**Published:** 2012-10-25

**Authors:** Valerii M. Sukhorukov, Daniel Dikov, Andreas S. Reichert, Michael Meyer-Hermann

**Affiliations:** 1 Helmholtz Centre for Infection Research, Braunschweig, Germany; 2 Frankfurt Institute for Advanced Studies, Frankfurt am Main, Germany; 3 Cluster of Excellence “Macromolecular Complexes”, Buchmann Institute for Molecular Life Sciences, Goethe University of Frankfurt am Main, Frankfurt am Main, Germany; 4 Mitochondrial Biology, Medical School, Goethe University of Frankfurt am Main, Frankfurt am Main, Germany; 5 Institute for Biochemistry and Biotechnology, Technical University Braunschweig, Braunschweig, Germany; Medical College of Wisconsin, United States of America

## Abstract

Mitochondria form a dynamic tubular reticulum within eukaryotic cells. Currently, quantitative understanding of its morphological characteristics is largely absent, despite major progress in deciphering the molecular fission and fusion machineries shaping its structure. Here we address the principles of formation and the large-scale organization of the cell-wide network of mitochondria. On the basis of experimentally determined structural features we establish the tip-to-tip and tip-to-side fission and fusion events as dominant reactions in the motility of this organelle. Subsequently, we introduce a graph-based model of the chondriome able to encompass its inherent variability in a single framework. Using both mean-field deterministic and explicit stochastic mathematical methods we establish a relationship between the chondriome structural network characteristics and underlying kinetic rate parameters. The computational analysis indicates that mitochondrial networks exhibit a percolation threshold. Intrinsic morphological instability of the mitochondrial reticulum resulting from its vicinity to the percolation transition is proposed as a novel mechanism that can be utilized by cells for optimizing their functional competence via dynamic remodeling of the chondriome. The detailed size distribution of the network components predicted by the dynamic graph representation introduces a relationship between chondriome characteristics and cell function. It forms a basis for understanding the architecture of mitochondria as a cell-wide but inhomogeneous organelle. Analysis of the reticulum adaptive configuration offers a direct clarification for its impact on numerous physiological processes strongly dependent on mitochondrial dynamics and organization, such as efficiency of cellular metabolism, tissue differentiation and aging.

## Author Summary

Mitochondria control energy production, initiation of cell death and several other critical cellular processes. Most often, they form a constantly reshaping tubular reticulum spread over the cytosol. Despite extensive knowledge of mitochondrial physiology, accurate description of their large-scale architecture is missing, partly due to substantial variability of reticulum geometries found in different cell types, and a capability for fast radical changes. We address this shortcoming with a mathematical model representing the organelle as a cell-wide dynamical network subjected to opposing actions of fission and internal fusion – processes known experimentally but not yet accurately specified. This opens a way for the quantitative characterization of the large-scale organization by showing how particular types of the internal dynamics can shape the reticulum into the whole multitude of configurations observed in biological studies. Further analysis reveals that for a specific value of tip-to-side fission/fusion rates the network should undergo a radical reorganization. Because of the high morphological sensitivity to minute changes in fusion or fission rates close to the critical point, cells can quickly adapt the mitochondrial operation and structure to their actual needs at a low expenditure of energy.

## Introduction

Whereas experimental biology provides important insights into numerous characteristics and protein complexes responsible for cellular physiology, understanding the functional properties of many organelles can best be achieved when cell-wide structural complexity is included. This is provided by mathematical models able to explore a wide range of spatial and temporal scales. In the present study we describe the spontaneous emergence of a cell-wide network of mitochondria as an effect of a small set of dynamical rules well characterized in biological studies.

**Figure 1:**
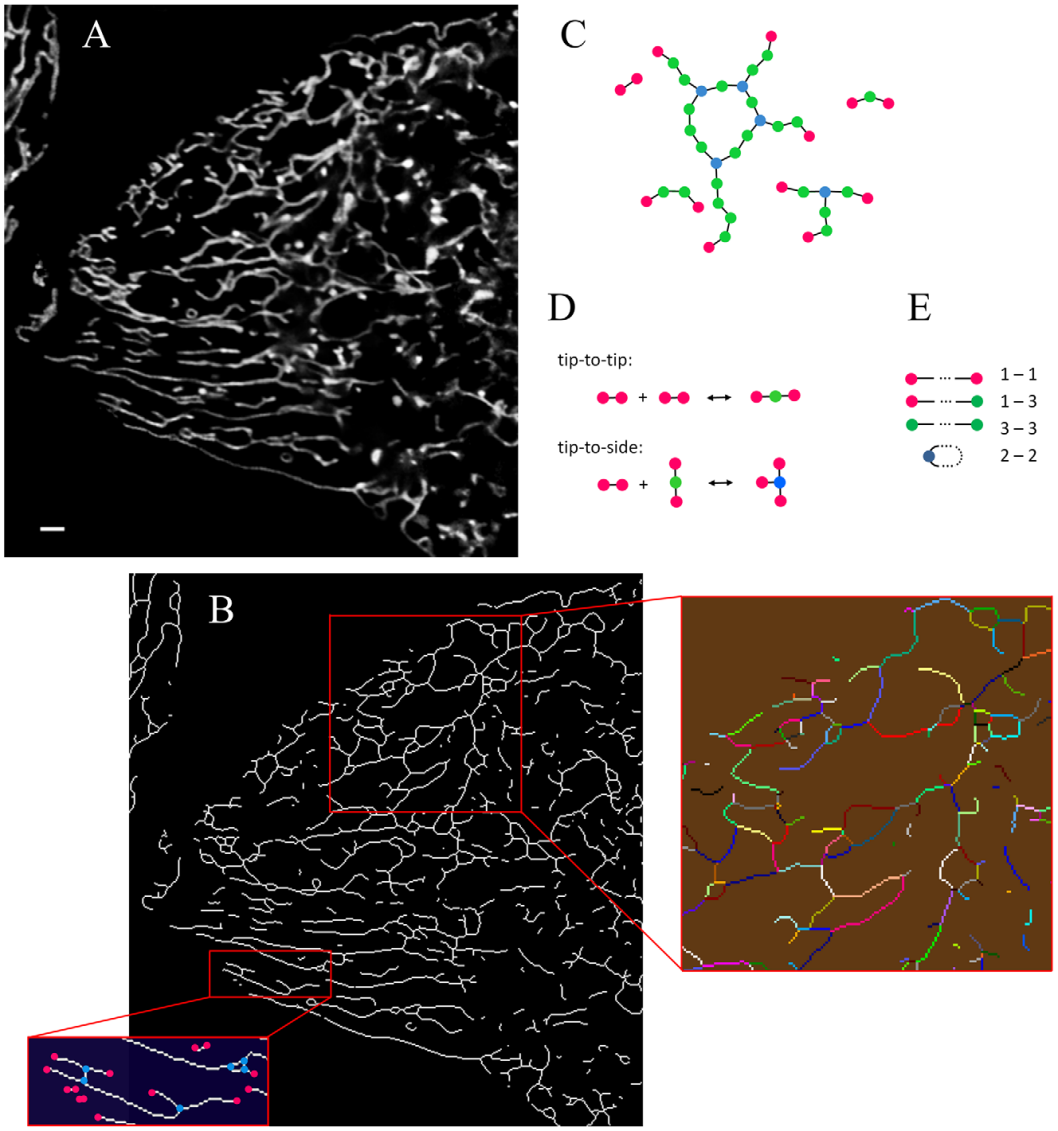
Large-scale structure of mitochondria. (*A*) Confocal micrograph (cross-section through cellular body) visualizing the mitochondrial network (scale bar is 1.3 µm) in a HeLa cell. (*B*) Result of thresholding and skeletonization of the reticulum in (*A*). *Upper rectangle*: decomposition of the reticulum into a set of interconnected linear segments. *Lower inset*: positions of detected degree *k* = 1 (*magenta*) and 3 (*blue*) network nodes. (*C*) Graph representation of the mitochondrial reticulum using three node types: *k* = 1 (*magenta*), 2 (*green*) and 3 (*blue*). The reticulum can be represented as a set of linear segments consisting of one or more edges (*black rods*) liking the nodes. (*D*) Two types of elementary network transformations comprising the reticulum fission/fusion reactions. (*E*) Segment types present in the network: (1-1) separate open-end segments, (2-2) separate loop segments, (1-3) surface and (3-3) internal segments of branched clusters.

Mitochondria are elongated intracellular organelles present in eukaryotic organisms ranging from yeasts to mammals. They are well known to produce the majority of cellular ATP, the universal form of energy required for most of cellular reactions and to be a critical checkpoint for the initiation of apoptosis. In the past few years mitochondria came into focus by ongoing discoveries of their central role in aging [1]–[3], ischemia [4], [5], development of cancer and common neurological and metabolic diseases [6]–[9] - processes dependent on complex interactions between mitochondrial subunits. In the cell they are present in variable numbers ranging from few to hundreds of entities, forming a dynamic partially interconnected reticular network which spreads over the whole cytosolic volume excluding the nucleus. The network architecture is rather diverse and flexible, is able to adjust itself on a time scale of minutes depending on the actual physiological condition, and is highly variable among different cell types. In the fully interconnected state, the network edges are approximately of cylindrical shape with a typical diameter a few hundred nm and highly varying lengths up to more than 10 µm [10]. The opposite extreme is the fully fragmented condition, where the mitochondria are roughly spherical vesicles with diameter similar to that of the aforementioned cylinders. In the majority of situations, a cell contains many networked clusters of varying sizes along with numerous individual mitochondria, thus representing an intermediate state between the two extremes (Fig. 1 *A*) [11], [12].

Quantitative description of this complex structure is currently not available, but it is thought to result from mitochondrial dynamics, governed by their constant intracellular motion along the cytoskeleton filaments and the ability to fuse and divide at varying positions at a time scale of minutes to hours [13]–[15]. The past years of experimental effort greatly enriched biochemical understanding of fusion and fission processes, which are accomplished via assembling the reaction-specific macromolecular complexes in mitochondrial membranes. Fission requires assembly of circular oligomers involving dynamin-like protein Drp1 attached to the outer membrane anchor Mff (in mammals) followed by GTP-dependent scission of the mitochondrial body perpendicular to the cylinder long axis. The fusion is performed by proteins (in mammals these include Mfn1, Mfn2 and Opa1) mediating tethering and subsequent connection of membranes surrounding the two input organelles and thereby generating a continuous body [16]–[19]. Despite variations in regulation and homology levels of the protein components, the above blueprint of the fusion/fission progression was found to be largely universal among different species [17]. Much less clear is what influence these elementary events have on the structural properties of the network as a whole and what their implications are regarding the functional efficiency of the cellular chondriome. Studies of other complex systems found that collective dynamics is often associated with phenomena not directly deducible from behavior of their isolated constituents [20], [21]. In relation to mitochondria, this distinction is potentiated by experimentally established interconnection between the organelle’s functionality and its reticulum configuration [22], [23 and Refs. herein].

Here, we report a whole-cell dynamical model of the chondriome able to capture both the recognizable variability of tissue-specific mitochondrial architectures and the network intrinsic flexibility in response to metabolic requirements, as observed experimentally [12], [24]. First, we examine the mitochondrial reticulum by image analysis of fluorescence microscopy in order to identify the key types of fission and fusion processes responsible for the reticulum connectedness. Then, the network structure and dynamics are recreated numerically using both mean-field ordinary differential equations and explicit stochastic agent methods. The evolutionary graph-based representation introduces well-definable concepts making possible accurate characterization of mitochondria in quantitative terms. Its in-depth analysis shows that (a) the two types of events - fusion and fission of mitochondrial bodies - are sufficient to explain the observed diverse organization of the mitochondrial reticulum, (b) the geometrical properties of the network are directly related and can be calculated from the frequencies of these two processes (and vice versa) based on a few assumptions well supported by experimental data, (c) the cellular mitochondrial reticulum should exhibit a percolation transition and (d) its plasticity can be well explained by the vicinity of its functional regime to the critical point.

## Results

### Nodes of degree three dominate the branching structure of the mitochondrial reticulum

The major characteristics determining collective properties of mitochondria are the ability of these elongated organelles to form tubular threads here referred to as segments and to further interconnect them into branched net-like structures extending over the volume of the cytosol within a cell. This continually changing reticular arrangement is clearly observable with fluorescence microscopy (Fig. 1 *A*). A comprehensive model of cellular chondriome must be based on a correct representation of essential dynamics of its components. Despite the overall agreement that the mitochondrial morphology is shaped by a delicate balance between fusion and fission [17], [25], different kinds of these processes are conceivable for the formation of three-dimensional tubular objects such as mitochondria. Which processes are actually active in living cells requires experimental elucidation.

Upon noting that the underlying dynamic behavior of mitochondria leaves a distinctive and stable footprint on the resulting network structure, the details of fission and fusion processes can be deduced from the morphological still image analysis. This is because different types of fission and fusion mechanisms (e.g. tip-to-tip in contrast to side-to-side fusion, etc.) do involve process-specific kinds of network nodes. Segmentation processing of confocal microscopy images was employed here to experimentally establish the types of fission and fusion events forming the mitochondrial reticulum based on its structural properties. Configuration of a spatial network is specified by the connectedness of its branches at node points, expressed as node degree *k* (number *k* of edges connecting the node to the rest of the network, Fig. 1 *C*) and does not explicitly involve branch lengths or other geometrical attributes [26], [27]. As a consequence, the mere presence or absence of nodes of degree *k*, rather than their relative abundance is sufficient in order to deduce a structure of the underlying dynamics and thus formulate a general model of the mitochondrial network and its evolution.

The main advantage of this procedure is circumvention of the problems related to explicit 4-dimensional reconstruction of the network, not sufficiently reliable yet (due to the limited resolution of the current microscopes in z-dimension, required to be less than the tubule radius, potentiated by the augmented noise resulting from fast scanning) [28]. With the structural aspects kept fixed by the experimental assessment, the consecutive mathematical model will explore all the possible geometrical configurations by varying the reaction rates. This standpoint mirrors the available evidence that organisms and tissues share fundamentally similar kinds of mitochondrial fission and fusion events, despite the differences in protein structure or compositional details of the corresponding molecular machineries [16].

The image processing algorithm identifies the network segments and their connection points (Materials and Methods, Fig. 1 *B*). Although some of the free mitochondrial ends (i.e. nodes of degree *k* = 1) in the pictures are artifacts arising from the apparent cutting a reticulum branch by confocal slicing, false higher degree nodes cannot be produced that way, leaving the branching points (*k*>2) unaffected. This enables the determination of the branching point types using single confocal sections per cell, therefore selecting the most advantageous focus position near the cover glass, where the reticulum structure is best resolvable (Fig. 1 *A*).

With a proper resolution, image voxel size has an effect on visible lengths of the mitochondrial tubules (and hence on apparent fraction of the bulk nodes *k* = 2) but not on the reticulum branching organization. Because the network structure is determined solely by the branching node types, quantitative image analysis is restricted to these only, without experimental evaluation of the bulk nodes contribution (inset at the bottom part of Fig. 1 *B*). On the other hand, in view of the importance of the nodes of degree two for the segment sizes and kinetics, these are included in the detailed model (see the next Section). There, relative contribution of all the node types will emerge dynamically from the fission/fusion activity.

Computational image analysis of mtGFP-harboring mitochondria in HeLa cells revealed an exclusive presence of apparent node degrees 1≤*k*≤4 (*n* = 7, see Materials and Methods for details). Complete lack of higher degree nodes indicates a very limited number of fission and fusion mechanisms involved in the network dynamics, in accordance with the small number of protein complex types known to perform these reactions [16], [24]. Relative abundance of nodes of different degrees is important, because it reveals geometrical constraints favoring the node formation of particular degree. So, the mechanisms capable of generating the branching node types *k* = 3 and 4 are dissimilar and can be enumerated explicitly. Assuming single node formation/disruption times much shorter than the characteristic time of fusion/fission kinetics, the degree 3 nodes result exclusively from interactions of a mitochondrial tip (*k* = 1) with a side surface (*k* = 2) (*a*), while the *k* = 4 nodes can only be created either by (*b*) fusion of a mitochondrial tip to an already existing branching site node (*k* = 3), or (*c*) by fusion of two bulk sites (*k* = 2) in organelles touching each other side-to-side. Thus, comparative abundance of nodes of degrees 3 and 4 reflects the relative impact of the above reaction types on mitochondrial structure. For example, the detection of a high fraction of crossing tubules (*k* = 4) would evidence for the significance of (*b*) or (*c*) for the network dynamics. Without the above assumption of fast elementary events, also interactions of three and four nodes would be conceivable, although experimental observations of such processes were not reported in the literature. Because of the expected small frequency of these higher-order interactions, the mathematical model utilizes events involving two nodes only: For example, generation of a *k* = 3 node from a triple of free tips would then involve a pair of tip-to-tip and tip-to-side fusions quickly following each other.

Because the thickness of confocal slices cannot be made less than approximately two times the typical mitochondrial diameter, some of the detected branching nodes represent false positive connections resulting from the overlay of two unrelated organelles along the microscope optical path. However, this artifact can be corrected for by estimating its probability from the known volume fraction of the cytosol occupied by mitochondrial bodies and their diameter.

In the examined micrographs, the occurrence of all apparent *k* = 4 nodes is 17.0±6.9% of the total branching points (Supplementary Material Table S1). The correction due to the aforementioned optical artifact reveals that at least 80% of those nodes with *k* = 4 result from the overlay of unrelated organelles. In contrast, for *k* = 3 the overlay contributes to 7% of the detected nodes, and for *k*<3 its effect is negligible. Hence, ∼96% of the actual branching points are of degree 3, while the fraction of *k* = 4 nodes is statistically insignificant. Although measurements performed here are focused at peripheral cellular regions with lower density of microtubules, the extreme dominance of the simplest branching type strongly indicates that the general ability of mitochondria to perform complex fusions of type (*b*) and (*c*) is extremely low. We conclude that the spatial network of mitochondria is essentially connected with branching points of degree 3 resulting from the tip-to-side fusion activity (*a*), consistent with reports using alternative methods [29]. Accordingly, the model discussed in the following is designed to exclusively exhibit nodes with 1≤*k*≤3.

### The model of mitochondrial network dynamics

We represent the mitochondrial reticulum with a graph (*nodes* linked by *edges*, Fig. 1 *C*) consisting of the following node types: free ends of mitochondrial segments (*k* = 1), bulk sites (*k* = 2) and branching points (*k* = 3). Network edges interconnecting the nodes define minimal (indivisible) constituents of the organelle and their length *l* introduces a spatial scale to the network. The graph formalism does not imply actual physical existence of discrete subunits of uniform size *l* inside the mitochondrial bodies, but incorporates in a formal way their divisibility potential. Physically, *l* corresponds to the average distance between the membrane-bound fusion or fission complexes projected on the mitochondrial body axis. Finite value of *l* reflects the fact that at any moment the chondriome contains finite number of the molecular machines potentially capable of the reactions. When fluorescently stained, such complexes are directly observable as discrete punctuate structures on the surface of mitochondrial membranes [30], [31]. Thus, the network edges reflect the maximal achievable fragmentation state of the chondriome, which disintegration into numerous vesicle-like tiny mitochondria is routinely observed experimentally and can be induced by strong downregulation of fusion or upregulation of fission [30], [32]. In the model, this potential for fission is provided by bulk nodes interconnecting the edges into linear threads and by branching nodes. On the cellular scale (>>*l*), the overall dynamics then leads to a structure (Fig. 1 *C*) similar to the real reticulum network as seen in Fig. 1 *A*. With the chondriome internally discretized by the edges and nodes, the graph-based formulation does not require any externally imposed coordinate system or lattice. As a whole, the network has *L* edges amounting to the total mitochondrial length in the cell. Because fission potential of the mitochondria is restricted by the finite number of the available molecular machineries, the discrete model reflects the biological situation better than continuous representation would do. In this organelle, the discretization parameter *L* representing the divisibility limit should be viewed as an important observable. For example, in the specific case of HeLa cell line, the total mitochondrial length as estimated by visual inspection corresponds roughly to 5000 µm. Because under the microscope mitochondria (diameter ≈0.2 µm) in the maximally fragmented configuration resemble spherical vesicles [30], [32], their diameter can be used as typical network edge length *l* = 0.2 µm, thus giving the value of the discretization parameter *L* = 3·10^4^ (≈5000/0.2). Keeping *L* as a free model parameter provides simple means for exploring the chondriome sizes relevant for different cell types.

Applying the principle of minimal complexity sufficient for the creation of the above network structure, in general we postulate two fusion and two fission types [33] represented as reaction processes on nodes *X_k_* (Fig. 1 *D*):

tip-to-tip fusion (*a*_1_) and fission (*b*_1_):
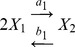
(1a)
tip-to-side fusion (*a*_2_) and fission (*b*_2_):
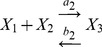
(1b)

Thus, the biological fusion or fission processes correspond to the network node transformations (Fig. 1 *D*) governed by specific evolution rules (Eq. 1). The graph-based, non-spatial formulation of the reticulum has the advantage that it allows studying structural development imposed by fusion/fission dynamics while omitting the detailed consideration of the underlying regulatory pathways, which may be organism or tissue-specific. By not discriminating biochemical protein species responsible for the corresponding reactions, the processes of Eq. 1 are set to account for their cumulative phenomenological effect by reproducing the observed spatiotemporal dynamics of the reticular structure, rather than mechanochemical description of inter-protein interactions. Still, taking into consideration that in mitochondria only one type of fission molecular apparatus is found experimentally, the description can be further simplified by assigning equal probability of a fission event per network edge: *b*_2_ = (3/2)*b*_1_ ≡ (3/2)*b*. This connection of fission rates for bulk and branching nodes via the number of participating edges reflects the fact that in both cases the fission event occurs by scission across a tubular body of the organelle.

Both the network dynamics and its equilibrium configurations result from intensities (*a*_[]_, *b*) of a few well-defined reactions, Eq. 1. The current study considers in detail a wide range of constant *a*_[]_ and *b*, which is sufficient for the characterization and explanation of the experimentally observed reticulum variability [11], [12], [16], [18], [30], [32]. Notably, interpretation of the reaction propensities may be extended by turning them into explicit functions of time- and concentration-dependent protein-specific kinetic processes. This would allow examination of the upstream regulatory mechanisms. However, such an expansion would involve inclusion of extramitochondrial biochemical pathways, exceeding the scope of this report.

The modeling framework proposed here (Eq. 1) can be utilized as long as the assumptions of (*a*) the network topology (1≤*k*≤3), and (*b*) absence of correlations between reaction events are fulfilled. This makes the model well suited for examination of mitochondrial networks, but would require major modifications in order to be applied to other cellular spatial networks such as endoplasmic reticulum. The condition (*b*) implies spatial isotropy and homogeneity inside the cell volume. Some anisotropy could arise in the very periphery of widely spread cells where microtubules may become arranged in bundles or preferentially oriented towards the cellular distal edge. So, care should be taken when applying the model to such cells. On very short spatial and temporal scale comparable with the kinetics of single motor proteins, mitochondrial fission and fusion may become also temporally correlated due to the influence of cytoskeleton. For example, two tips of mitochondria created by a recent division event but still attached to the same microtubule would have higher immediate propensity for fusion. However, such a deviation can occur only if the density of the cytoskeleton is sufficiently low, e.g. in the very periphery of the cell. In the bulk of the cytosol, frequent transitions between the fibers resulting from the high density of the cytoskeletal mesh common for the eukaryotes are expected to average the effect of single filaments out, leading to a fast decay of such correlations. This supposition was checked and confirmed by control simulations using an extended model where the mitochondrial network elements were assigned spatial positions by connecting them to explicit mesh of cytoskeletal fibers (data not shown). For configurations and densities of microtubules typical for central cellular regions, the prevailing majority of mitochondrial segments were found in the immediate proximity to several differentially oriented fibers simultaneously, leading to their fast reorientation. Consequently, the well-mixed environment is sufficient for the present investigation, in which the extremely short time scales are not considered.

Accounting for limited diffusivity in crowded cytosolic environment was shown to be important for accurate representation of molecular chemical systems due to their impact on effective kinetics relative to dilute conditions [34]. Mitochondria are much larger objects and are driven actively by motor proteins, but their mobility can still be affected by hindrances and other distortions commonly modeled by variations in diffusion coefficients. This kind of influence is accounted for in the current graph-based representation too, although a different approach is utilized. The dynamics of mitochondria is governed here by node transformation rates, and thus the diffusion in physical space is not explicitly implemented. Instead, the actual effective values for fusion/fission rates are taken from experimental measurements performed in living cells [15], which overall account for all known and unknown factors affecting the dynamics, without discrimination between those internal and external to mitochondria. This corresponds to a well-mixed approximation and allows the model to reproduce the architectural build-up of the reticulum while avoiding ambivalences related to still scarce experimental data on complex motility patterns of mitochondria subject to multiple regulatory mechanisms. Importantly, the framework of dynamic quantitative graph introduced here can be utilized for an upgraded model where coordinates, velocities, and/or external forces are assigned explicitly to the network constituents.

In addition to fusion/fission dynamics, the mitochondrial reticulum is subject to ongoing biological renovation in the course of import/export of material accompanied by organelle degradation due to selective mitochondrial autophagy (mitophagy). Carried out by protein molecules quite uniformly distributed over the mitochondrial body, the import/export is not known to influence or to be sensitive to the network structure. The total mitochondrial mass varies synchronously with the cell mitotic cycle or as a response to signals changing gene expression patterns – both processes much slower than the fusion or fission [15], [18], [25]. Hence, value of *L*, the parameter accounting for the organelle size is assumed time-independent here. The mitophagy potentially could influence the size distribution by preferentially depleting very small clusters because it is limited to small (∼1 µm) organelles. However, its impact on the mitochondrial architecture is negligible under physiological conditions because of the differences between time scales characteristic for the autophagy and the fission/fusion: while the newly imported or synthesized mitochondrial material was experimentally found to persist there for up to a few weeks, the mitochondrial motility is sufficiently fast to adapt the network conformation on the time scale of minutes to hours [13]–[15], [35]–[37]. Hence, the reticulum will be considered here well-equilibrated from the material turnover point of view. Notably, the model formulation allows for an extension explicitly incorporating autophagy into alternative implementations specifically targeted for investigation of longer durations. This can be useful if properties exceeding the mitochondrial structural organization, such as quality control mechanisms, were to be examined in detail [2], [38].

For the following, a mitochondrial *segment* is defined as one or more network edges connected only through bulk sites (nodes of degree 2), and a *cluster* as a detached set of interconnected segments possibly containing also branching nodes.

### Deterministic description links morphology to the fission and fusion rates

When expressed in the numbers of nodes per cell *x_k_*, Eqs. 1 translate into a system of differential-algebraic equations governing the network dynamics:
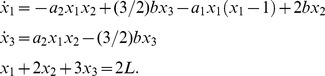
(2)


The terms on the right-hand side of the first two of Eq. 1 represent the node kinetics due to each of the fission and fusion processes. For example, consumption of mitochondrial tips (*k* = 1 nodes) in the course of tip-to-tip fusion -*a*_1_ *x*_1_(*x*_1_-1) is a product of the total number of node pairs potentially capable of fusion *x*_1_(*x*_1_-1)/2 and the fusion propensity for a pair 2*a*_1_. The last of Eq. 2 reflects conservation of the mitochondrial mass in the cell expressed as total number of edges *L*. Technically, it restricts the system phase space **x** ≡ (*x*_1_, *x*_2_, *x*_3_) to a plane which position and orientation are uniquely specified by *L* and the network structure **k** ≡ (1,2,3) respectively. While changing the applied discretization *L* of the model merely shifts the phase plane parallel to itself, relative proportions of the nodes and thus the state of the reticulum as a whole are well defined by the parameterization parameters (*a*_[]_, *b*). Alteration of the reticulum discretization *L* corresponds to a different expression level of fission/fusion complexes without changing their relative abundance, while the values of *a*_[]_ and *b* reflect independent activities of each of the network transformation processes.

The steady state of the system is a function of the ratio of fusion and fission rates *c*_1_ ≡ *a*_1_/*b* and *c*_2_ ≡ *a*_2_/*b* rather than the rates themselves:
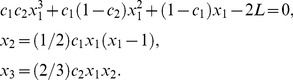
(3)


**Figure 2:**
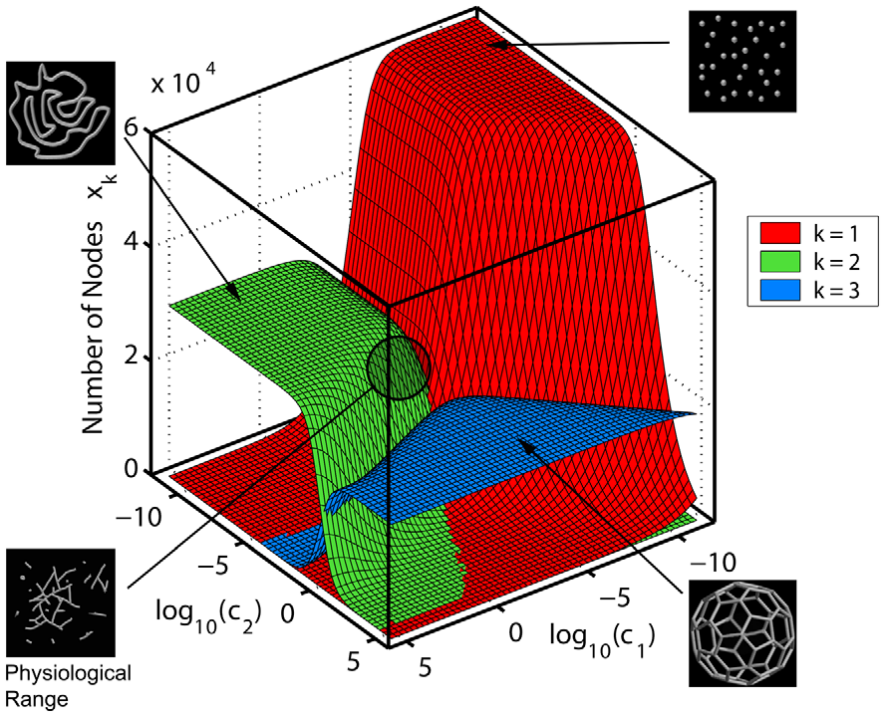
Steady state solutions (**Eq. 3**) of differential-algebraic model of the mitochondrial reticulum (**Eq. 2**). Numbers *x_k_* of network nodes of degrees *k* = 1,2,3 are plotted as a function of relative intensities of fusion and fission for a mitochondrial reticulum of size *L* = 3·10^4^ approximately corresponding to a HeLa cell line. A unique reticulum configuration characterized by the number of mitochondrial tips *x*_1_ (*red*), bulk nodes *x*_2_ (*green*), and branching sites *x*_3_ (*blue*) corresponds to each value of the ratio between fusion and fusion rates for tip-to-tip (*c*_1_) and tip-to-side (*c*_2_) processes. Schematic drawings of representative network configurations are shown in the insets, along with their approximate location in the parameter space. Unlike the fragmented or hyperfused networks resulting from disproportionate fusion and fission activities, in the physiologically relevant parameter range the reticulum configuration is heterogeneous and especially sensitive to changes in *c*_1_ and *c*_2_.

A unique set of real non-negative solutions **x** describing the network state corresponds to each triple of parameters (*c*_1_, *c*_2_, *L*). Fig. 2 exemplifies the steady-state solutions (Eq.3) for a particular chondriome size corresponding to HeLa cell line (*L* = 3·10^4^, see previous Section) and a wide range of (*c*_1_, *c*_2_). In the extreme of infinitely strong tip-to-side fusions *c*_2_→+∞, the network tends to a fully connected “crystal” **x** → (0, 0, (2/3)*L*) consisting of branching sites only. Because in this regime the reticulum has no other node types, its geometry is insensitive to *c*_1_. The opposite case (*c*_2_ → 0) allows for many different configurations specified by the ratio of tip-to-tip fusion and fission rates *c*_1_: the set of possible states is flanked here by a fully fragmented **x** → (2*L*, 0, 0), *c*_1_→0 and a single loop **x** → (0, *L*, 0), *c*_1_→+∞. Reticulum states corresponding to some of these extremes were experimentally induced by artificial manipulation of mitochondrial fission or fusion activities [30], [32]. However, physiologically the most relevant parameter range is fairly narrow (see below), with all *x_k_* being far from saturation (Fig. 2).

The deterministic description of the mitochondrial geometry (Eq. 2) establishes a well-defined analytical connection between the molecular biochemical parameters (*a*_[·]_, *b*, *L*) and network-wide structural variables like the average numbers of nodes *x_k_*, mean segment length for non-loop segments 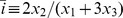 and total number of segments (*x*_1_+3*x*_3_)/2 comprising the reticulum. These represent the simplest quantitative measures of the chondriome architecture. They can be determined experimentally from the analysis of high-resolution cell reconstructions recorded with sub-diffraction microscopy, which is currently being extensively developed and shall become available in biological laboratories [39]–[41]. This type of recordings could also go beyond the mean values and measure among others the probability distributions of mitochondrial segment lengths and cluster sizes (discussed below). In addition to a more complete characterization, the latter variables may prove critical for clarification of key features of this organelle by revealing potential effects of stochastic fluctuations on mitochondrial operation and homeostasis (see the Section “Percolation phase transition in the mitochondrial reticulum” below).

### Agent-based dynamics for the study of chondriome stochastic characteristics

In order to obtain a more detailed insight into the expected mitochondrial reticulum architecture, an agent-based stochastic simulation [42] of the same system was developed. As was verified by investigation of the potential fusion points in a setting with explicit representation of the cytoskeleton (data not shown), for the long-term evolution a non-spatial approximation is justified inside the majority of the cytosolic volume. Hence, a stochastic model was developed, where a set of *L* reactant objects corresponding to the network edges is submitted to the processes of fission and fusion as above (Eq. 1), i.e. between random nodes without explicit positioning in space. Reaction events and timings are put under the operation of the Gillespie algorithm [43] where nodes participating in a particular event are chosen randomly with equal probability among nodes of the same type. In the absence of detailed biochemical data on fission and fusion rates, the simulation parameters were adjusted to reproduce the experimentally observed average frequencies of fusion and fission events ≈0.25 (cell·sec.)^−1^ [15]. Explicit representation of the cellular mitochondrial system within the agent model allows for a comprehensive insight into the network characteristics surpassing the mean-field approximation of the deterministic description above. Hence, the following discussion is focused on the stochastic properties of the reticulum expressed in terms of statistical distributions.

### Tubular segment lengths are well approximated with a geometrical law

Alternatively to the node-based description, the discussed network can also be viewed as a set of four types of (partially) connected segments, discriminated by degrees of the two end nodes (Fig. 1 *E*). The network state is then characterized by segment numbers of each of the four types: separate open-end segments 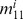, separate loops 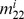, as well as surface 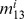 and internal 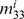 segments of branched clusters. Index *i* denotes the segment length measured in edges.

**Figure 3:**
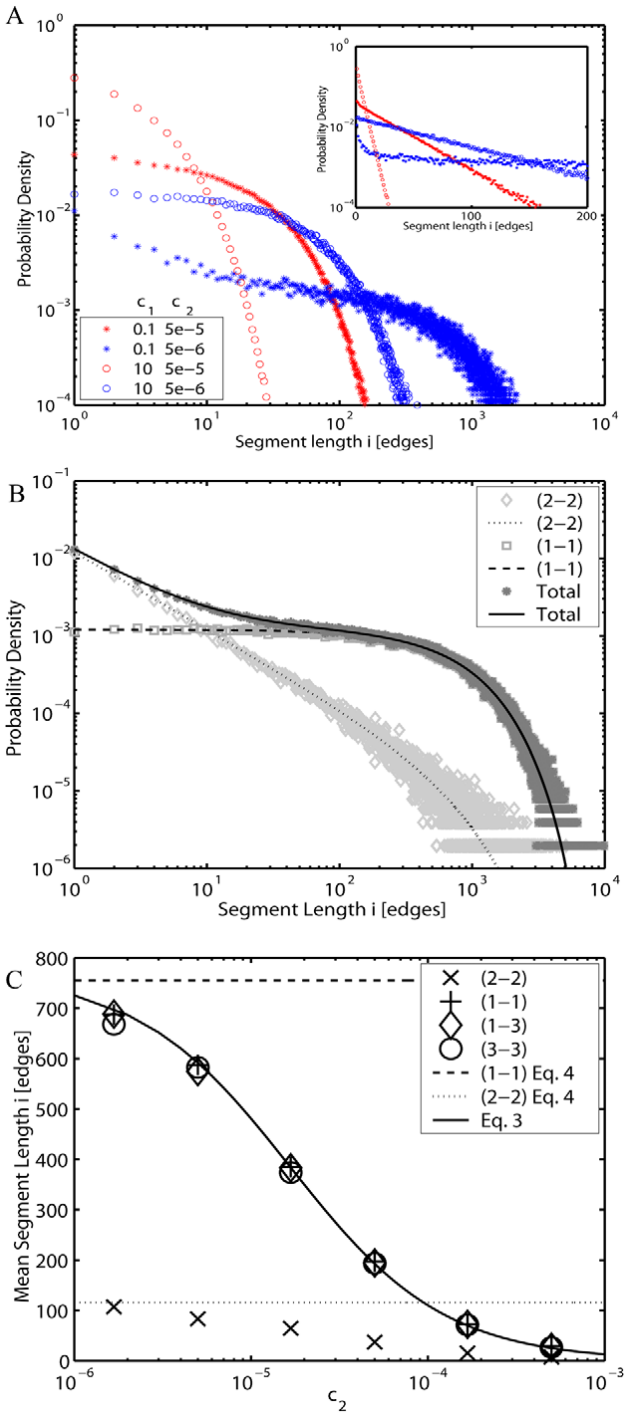
Chondriome segment lengths (*L* = 3·10^4^). (*A*) Examples of segment length distributions for different values of tip-to-tip and tip-to-side fusion/fission rates *c*_1_ and *c*_2_ respectively (*stars*: *c*_1_ = 0.1; *open circles*: *c*_1_ = 10; *red color*: *c*_2_ = 5.0·10^−5^; *blue color*: *c*_2_ = 5.0·10^−6^) in the agent-based representation. The same data plotted using semi-log scaling (*inset*) highlight deviations from the geometrical decay in short segments due to influence of loop structures. (*B*) Distributions for loops (*light gray diamond*), open segments (*gray square*) and total (*dark gray star*) for the simplified network (*c*_2_ = 0) as calculated by the stochastic algorithm, compared to the exact analytical results: *Dotted and dashed lines* are contributions from loops and open segments, respectively, according to Eq. 5 (after normalization). (*C*) *Markers* - mean values differentiated by segment types as determined by the agent-based simulations: (1-1) - *plus*; (1-3) - *diamond*; (2-2) - *cross*; (3-3) - *circle*. *Solid line*: 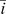 from the deterministic model. *Dotted* and *dashed lines*: Limiting values at *c*_2_ = 0 (Eq. 5) for sizes of loops (2-2) and open-ended (1-1) segments respectively.

Using the agent-based model, we find that with a good accuracy the steady-state distributions of mitochondrial segment lengths (Fig. 3 *A*) can be expressed as a superposition of two qualitatively different, fast and slow decaying, terms with their relative strength being strongly dependent on *c*_1_ and less on *c*_2_. The two components can be examined analytically by considering a simplified network where tip-to-side fusions are switched off (i.e. *c*_2_ = 0). As the degree of nodes is restricted here to *k* = 1 and 2, such a network consists of (1-1) and (2-2) segment types only, and equations governing their dynamics can be formulated explicitly. The segment numbers of length *i* 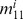 and 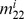 are derived by taking into account all possible transitions between segment populations:
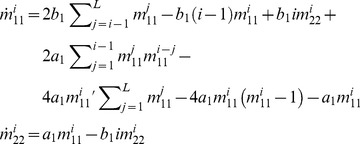
(4)


Here the prime under the sum denotes omission of the term *j* = *i*. The consecutive terms on the right-hand side of the Eq. for 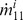 correspond to (1) creation of the segment resulting from fission of longer segments, (2,3) disruption and creation due to fission of same-size open-end segments and loops respectively, (4) formation upon fusion of shorter segments, and (5,6) disruption resulting from fusion to different and same size segments, respectively, and (7) formation of loops. Expressions for the steady-state segment length distributions in this network reveal unambiguous distinction in length distributions between loops and open-ended segments (Fig. 3 *B*):
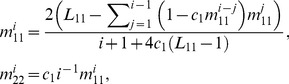
(5)with 
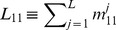
 being the total number of open-ended segments. While Eq. 5 takes into account finite-size effects, as anticipated in real cells, for an idealized infinite system one would expect a geometrical distribution of 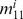 because of the uniform probability density for a segment production or disruption over bulk nodes. On the other hand, the sizes of loops are governed by power-law. As a result, among the small segments the number of loops strongly exceeds the amount of the open-ended segments, while for the long segments the proportions are reversed.

In the general network (*c*_2_>0), the tip-to-side fusion and fission generates branched clusters consisting of variable segment numbers. The possibility of transitions between them induces a mild deviation from Eq. 5. Still, for all segment types other than disconnected loops (2-2), the geometric distribution 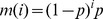, where *p* is the probability of a segment end provides a good approximation to the agent-based result. The mean length of segments 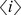 can be estimated from the number of nodes introduced in the deterministic model 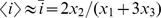 (Fig. 3 *C*, *solid line*) and is therefore related to fusion and fission rates through Eq. 3. In the equilibrated system, 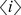 is essentially equal among all non-loop segment types or cluster sizes (Fig. 3 *C*). Importantly, due to the strong decrease of 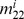 at lower *i* (cf. Eq. 5, Fig. 3 *B*), loop segments amount to only a few percent of the total mitochondrial mass (Fig. 3 *C*, *crosses*), hence the mean length 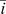 can be viewed as a good network-wide measure of mitochondrial reticulum structure (Fig. 3 *C*, *solid line*).

The geometric law of segment lengths is a direct consequence of the balance between opposing actions of fusion and fission. The pattern results from the positional independence of fission sites along the body of a mitochondrial tube, accompanied by the equal chances of network nodes to become involved in a fusion event (i.e. with probability being independent from the lengths of the segments). This corresponds to the dynamic behavior of a well-mixed system, as expected upon a cell-wide equilibration of the chondriome resulting from the permanent motion of individual mitochondria along the cytoskeleton filaments [13], [14].

### Sizes of disconnected chondriome parts correspond to the negative binomial distribution

**Figure 4:**
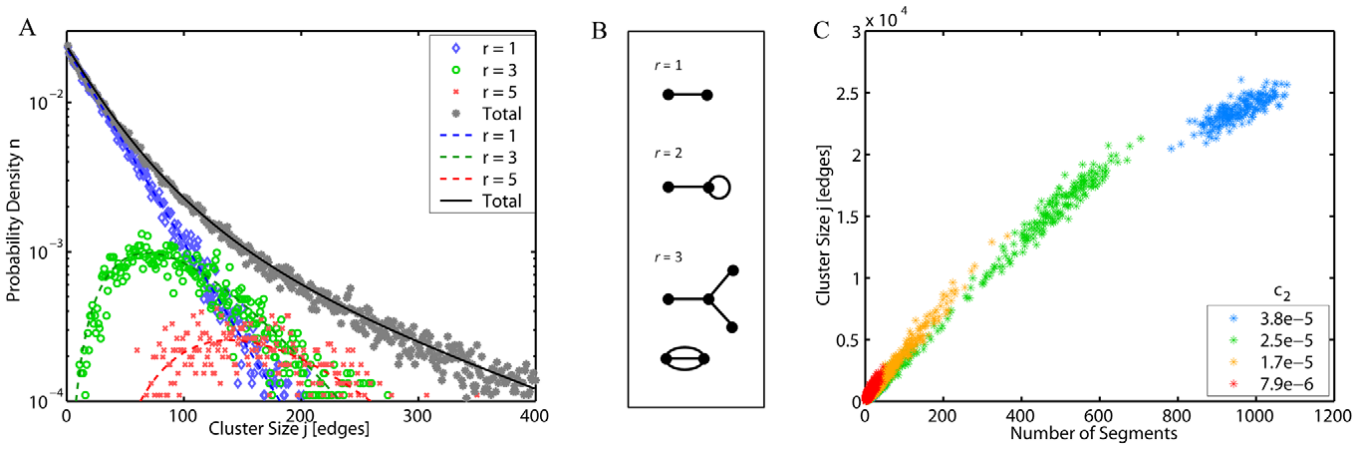
Sizes of disconnected mitochondrial clusters. (*A*) Distribution of cluster sizes in the vicinity of percolation transition (*c*_1_ = 0.1, *c*_2_ = 2.2·10^−5^, *L* = 3·10^4^) from agent-based simulation (*stars*) superimposed with *n*(*j*), Eq. 7 (*black solid line*). Contributions from 1-segment (*blue diamonds*, *r* = 1), 3-segment (*green circles*, *r* = 3) and 5-segment (*red crosses*, *r* = 5) clusters are shown separately together with corresponding fits, Eq. 6 (*colored dashed lines*). (*B*) Typical configurations of clusters for *r* = 1, 2 and 3 segments (for clarity, bulk nodes are not shown). (*C*) Scattered plots of cluster size *j* vs. number of segments per cluster for different *c*_2_ (*legend*) and *c*_1_ = 10, *L* = 3·10^4^. Note the elevated spread of cluster sizes in the vicinity of the critical point (*yellow*, *green*). The scatter slopes for various *c*_2_ reflect change in the mean segment length.

For general values of *c*_1_ and *c*_2_ the system contains numerous segment clusters of variable sizes. Because the lengths of segments in a cluster are geometrically distributed independent random variables, the sizes *j* of clusters (i.e. the total number of network edges within a cluster) with *r* segments (Fig. 4 *B*) in the agent-based system conform (Fig. 4 *A*, *colored markers*) to the negative binomial distribution (Fig. 4 *A*, *dashed lines*)
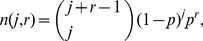
(6)where *p* is the probability of a segment end. The integral size distribution of all clusters in the system
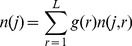
(7)involves weighting by the number of *r*-segment clusters *g*(*r*), which has no known closed form expression for this network geometry. Yet, upon using the agent-simulated *g*(*r*) together with Eq. 6, one recovers *n*(*j*) numerically (Fig. 4 *A*, *black solid line*, *grey stars*).

Variations in cluster sizes *n*(*j*) expected in a single cell are illustrated in the scatter plot of cluster sizes vs. number of segments in the cluster, for a set of different tip-to-side fusion rates *c*_2_ (Fig. 4 *C*). The dependences can be viewed as linear, with their coefficients (slopes of the scatter) corresponding to well-defined mean segment lengths 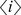 discussed above. Increase in *c*_2_ leads to the conversion of bulk sites into branching sites and the resultant gradual reduction of 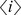 (see also Fig. 3 *C*) accompanied by a higher segment number. Much less intuitive are *c*_2_-dependent nonlinear changes in variance (spread) of the cluster sizes (Fig. 4 *C*) discussed in the following.

For the simplified dynamics considered above (*c*_2_ = 0, Eqs. 4,5), *n*(*j,r*) would be reduced to a mixture of geometric and power laws (see previous Section), because such a network consists from single-segment clusters only.

### Percolation phase transition in the mitochondrial reticulum

**Figure 5:**
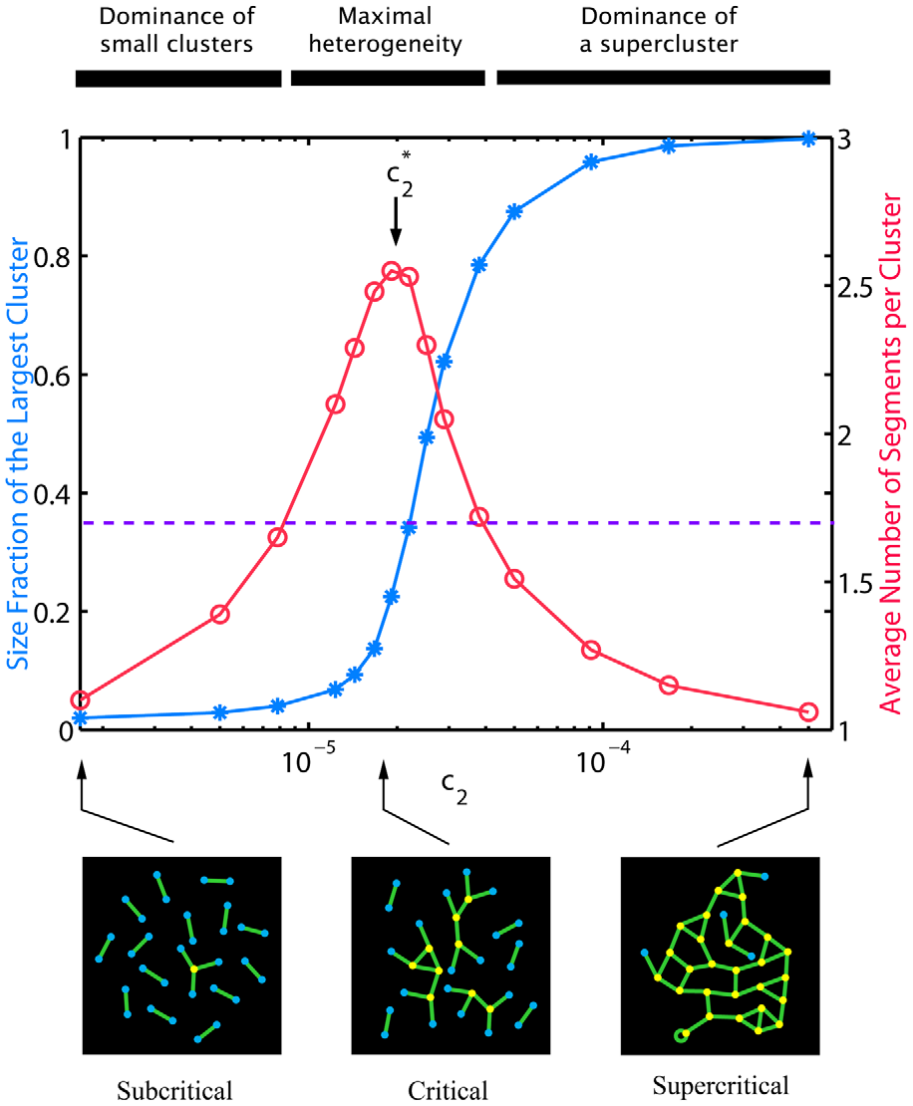
Percolation transition in the mitochondrial network for *c*_1_ = 0.1, *L* = 3·10^4^. (*Upper part*) Fractional size of the largest cluster in the system (*blue stars*) and the corresponding average number of segments per cluster in non-giant clusters (*pink circles*). The position of the critical point (*arrow*) corresponds to approximately 30% of largest cluster size fraction. For comparison, the experimentally determined largest cluster size fraction was 35±20% (*dashed line*). (*Bottom part*) Qualitative differences between subcritical, critical and supercritical regimes of the reticulum (*c*_2_ = 1.7·10^−6^, 1.9·10^−5^ and 5.0·10^−4^ respectively, which corresponds to the ratios of end nodes (*blue*) to branching nodes (*yellow*) *x*_1_/*x*_3_≈31, 2.7 and 0.14 respectively): Although numbers of network edges (overall mitochondrial mass) are the same for the three configurations, the cell-wide connectivity is only possible above the critical value of tip-to-side fusion/fission ratio *c*_2_≈2.0·10^−5^ (for clarity, bulk nodes are not shown and segments (*green*) are sketched of equal length).

The presented reticulum model (Eq. 1) exhibits a percolation phase transition in parameter *c*_2_ (tip-to-side fusion/fission ratio). In the thermodynamic limit of infinitely large network, the transition over a percolation threshold would correspond to an abrupt formation of a giant (percolating) cluster. At critical value 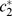 a qualitatively new structure arises - a global cluster created from otherwise unconnected chondriome components spreading over the remote intracellular regions (Fig. 5, *blue stars* and *schemes*). In the finite network of a real cell the abrupt change of the network order is smoothed in the vicinity of the critical point, but rapid emergence and then dominance of the giant cluster for *c*_2_>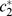 is manifested when the fractional size of the largest mitochondrial cluster is plotted as a function of *c*_2_ while keeping *c*_1_ constant. Continuous (second order) phase transitions are marked by a peak in size fluctuations or other susceptibility measures at the point of criticality [44]. In the modeled reticulum the peak is visible as a sharp change in variability of non-percolating cluster sizes, e.g. expressed as the average number of segments per cluster (Fig. 5, *pink circles*). In a scatter plot, the rapid rise in cluster size fluctuations near the transition point is undoubtedly noticeable as elevated spread in sizes of individual clusters comprising the cellular chondriome (Fig. 4 *C*, *yellow* and *green* versus *red* and *blue*).

In contrast, no phase transition is found in tip-to-tip rate *c*_1_, where the critical point should be expected only at *c*_1_ →+∞, similar to a one-dimensional percolation problem on the lattice [44]. This result can be intuitively understood: While increase in tip-to-tip fusion rate *c*_1_ merely raises the average segment length, the tip-to-side fusions (*c*_2_) interconnect the segments among themselves and thus promote the formation of a global networked structure. Although from a mathematical perspective the predicted criticality is not surprising given the network topology assumed in the model, its relevance for the structure of mitochondria may have long-standing implications for cellular organization and functionality.

The predicted phase transition implies two distinct classes of mitochondrial structures: a subcritical network consisting of a relatively uniform set of multiple disconnected mitochondria, as opposed to a supercritical one characterized by the presence of a dominant giant cluster accumulating the majority of the mitochondrial mass, accompanied by a few much smaller satellite mitochondria (Fig. 5 *bottom part*). On one hand, from the experimental perspective, because only a relatively narrow range of *c*_2_ values corresponds to the transitional regime, the great majority of cells under unselective conditions could be expected to be found far from the transition point 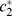. On the other hand, frequent observation of intermediate chondriomes would indicate an underlying regulatory or self-tuning mechanism selectively promoting quasi-critical configurations.

Both highly fragmented and highly interconnected structures were induced experimentally in several cell types with pharmacological treatment severely shifting the fission/fusion balance [30], [32]. However, quantitative experimental investigations of the chondriome's undisturbed geometrical organization are, to our knowledge, not yet available. Here, we preliminarily examined the relevance of the predicted percolation transition for chondriome structure by measuring relative sizes of disconnected mitochondrial clusters in confocal images analyzed in the first Section (see also Materials and Methods). We find that in a standard mammalian cell line (HeLa, Supplementary Material Table S1), the largest mitochondrial cluster comprises 35% of the total visible reticulum (Fig. 5, *dashed line*), a fraction far from both the high-end and low-end saturation echelons expected for single-phase configurations (Fig. 5, *blue stars*). This value is accompanied by high (st. dev. 20%) diversity among cells, pointing to an elevated level of fluctuations in the cluster sizes. This result is further supported by the examination of relative sizes of mitochondrial clusters belonging to the same cell (data not shown). Both parameters indicate that under the normal physiological conditions the mitochondrial network may operate near the percolation transition point. However, due to small sampling of this preliminary measurement, currently only a compatibility with this interpretation can be stated. A more rigorous validation of the percolation transition predicted here will be achievable, when precise tuning of fission and fusion frequencies along with three-dimensional reconstruction of mitochondrial networks in living cells becomes biologically possible.

## Discussion

Growing experimental evidence indicates the fundamental interdependence between mitochondrial metabolic activity and its network structure [22], [23 and Refs. herein]. Yet, until now, the theoretical analysis of this vital organelle was either focused on its biochemical and electrophysiological aspects [45]–[47], or was reducing its architecture to a set of linear objects [48]. The examples of other complex systems indicate, that the manifestation of collective properties of the mitochondrial net should be critically dependent on proper representation of its structure and dimensionality [20], [21], [44], [49], requiring a cell-wide multiscale formulation. The current work examined the chondriome organization experimentally and introduced its network-based mathematical representation. This led to a detailed insight into the organelle, emerging as a mesh of tubular segments interconnected into larger flexible clusters able to reach distant cellular regions.

We find that fusion and fission dynamics should lead to a branched reticulum of tubules which lengths are well approximated by a geometric law and which mean size in equilibrium is determined by relative rates of these processes (Eq. 3). The whole network consists of disconnected clusters of such tubules. The cluster sizes are well approximated with a superposition of negative binomial distributions (Eqs. 5, 6). Notably, the distribution shape is distinctly convex (Fig. 4 *A*), featuring numerous tiny clusters coexisting along with a few relatively large entities. This property is expected to promote the experimentally observed disposal of damaged mitochondria by cellular autophagosomes [2], [50]–[52]. One reason for this is that mitochondrial dysfunction was shown to inactivate the mitochondrial fusion machinery, consequently leading to smaller mitochondrial entities [53], [54]. In addition, prevalence of small clusters present in the network supports the formation of autophagosomal bodies, which are not able to engulf objects larger than a few µm in mammals [37], [55]. Moreover, smaller clusters are expected to exhibit high statistical variance in functional efficacy facilitating the determination of removal candidates based on the inner membrane potential gradient or similar markers. In this way, the viability of an organism could be optimized by maintaining homeostasis of high-quality mitochondrial material on the whole-cell level [38].

Gradual disruption of this quality control e.g. as a result of natural aging is known to coincide with simultaneous rearrangement of mitochondrial reticulum structure due to alteration of fission and fusion rates [2], [15], [51]. By experimentally manipulating mitochondrial dynamics, the network reorganization was found to be sufficient for the induction of a substantial slowing down of the aging process [1]. The cluster size distribution predicted here offers a quantitative explanation for these and similar observations related to the strong dependence of mitochondrial structure and function [23].

To what extent the ongoing network dynamics is able to smooth out the differences constantly arising from diverse functional activity in distant parts of the chondriome? On a shorter time scale (∼minutes), the ongoing fission prevents complete homogeneity of the mitochondria over the cell body. Inside the mitochondrial clusters, additional equilibration results from molecular diffusion along the organelle tubules [56]. The geometric (exponential) distribution of the segment lengths predicted here is characterized by a high variance, with very long tubules connected to multiple shorter ones. It would be interesting to check what biological implications this diversity has for the organelle performance. For example, taking into account that key mitochondrial proteins in the inner membrane tend to compartmentalize while their diffusion is very slow [56]–[59], the presence of very long segments could significantly hinder equilibration of compositional differences between reticulum branches.

The balanced fusion and fission dynamics as in Eq. 1 leads to a network capable of a phase transition, i.e. that possessing two qualitatively dissimilar organizational modes. The critical transitional region lays in a narrow range of tip-to-side fusion/fission rates (*c*_2_, Fig. 5) where the reticulum structure is able to rapidly change its configuration, i.e. is very susceptible to the proper balance of these opposing processes. An experimental examination of the chondriome structure in HeLa cells reveals that its geometry corresponds to the transitional regime, characterized by the maximal heterogeneity in sizes of the network subcomponents. If this result is confirmed for other cell types, this would imply that under normal physiological conditions the cell has to maintain the fission and fusion rates quite precisely matched, despite the necessary flexibility in other parameters. The tight positioning inside the narrow transitional region rather than in one of the numerous configurations away from the critical point would induce questions about factors responsible for such a specific arrangement. Their assessment exceeds the scope of the current study, whilst the details of the network dynamics and architecture examined here could serve as an important counterpart. Similar combinations of high susceptibility and phenotypic robustness were found in other complex adaptive systems [60]–[62].

Independently from the underlying factors, it would be, indeed, advantageous for a cell to operate in the vicinity of the critical point because here the mitochondrial reticulum can be reconfigured with minimal energetic and temporal cost. Examples for such transformations include the quick and radical mitochondrial fragmentation upon activation of the apoptotic cascade or in the course of mitosis, where it is essential for a proper partitioning of the organelle between the daughter cells [63], [64]. Furthermore, high susceptibility of the mitochondrial network in the critical regime to small changes of the branching parameter naturally generates a high diversity between cells. Well known experimentally, it was often discarded as inessential intercellular noise amid a homogeneous population. However, this view is now changing due to recent observations connecting cellular structural aspects to its functional and gene expression patterns [65]. For example, in the course of organogenesis, the narrowly positioned regime of elevated flexibility can facilitate the tissue-specific reticulum alteration. With such a mechanism in place, during the differentiation phase cells can attain chondriomes best suitable for specialized energetic needs, inducing the variability of mitochondrial geometries found in different tissues and cell types [11].

In conclusion, the proposed model explains the self-organization of the chondriome into a dynamic network and its operation as a cell-wide adjustable construct, which large-scale characteristics provide the necessary connection between microscopical biochemical parameters and qualitative features central for the functionality of living cells. The predicted size distributions of segments and clusters are easily interpreted in physiological terms and verifiable by corresponding experiments.

## Materials and Methods

### Cell culture, transfection and imaging

HeLa cells were grown in Dulbecco Minimum Essential Medium (Sigma), supplied with 10% FCS (PAA) and 1% Penicillin Streptomycin (Sigma). Effectene (Qiagen) was used for transient transfection in 3.5 cm Petri dishes (IBIDI) according to the manufacturer's instructions. mtGFP experiments were performed 24 h after transfection. 4-(2-hydroxyethyl)-1-piperazineethanesulfonic acid (HEPES, 10 mM final concentration) was added to the medium 1 h before measurements. Live cell imaging was done at 37°C using Nikon Eclipse TE2000-E microscope. A small pinhole ensured that the thickness of confocal slices did not exceed 800 nm. In order to reduce the effects of perinuclear region irregularities, the system was focused to a fraction of the intracellular volume adjacent to the Petri dish bottom.

### Image analysis

Raw confocal images of HeLa cells (Fig. 1 *A*) with fluorescently visualized mitochondria were subjected to digital analysis designed to determine the reticulum network structure. As the first step, the images were thresholded and skeletonized after contrast optimization. The threshold position was chosen for each image such that no mitochondrial signal was lost while the background was cut off. Subsequently, the binary maps representing spatial graphs of the reticulum resulting from the skeletonization operation (Fig. 1 *B*, *main field*) were treated with a segmentation algorithm designed to identify and statistically analyze mitochondrial linear segments and branching points interconnecting them into clusters. An input skeletonized image consists of white pixels (1) on the background (0) (Fig. 1 *B*), the former classified as nodes of the mitochondrial network. Two white pixels were considered forming a connected graph if they were found adjacent to each other by applying an 8-connectivity criterion [66]. Degrees *k* of the resulting network nodes were calculated as the number of neighbors adjacent to the corresponding white pixel and, if appropriate, corrected to account for oversampling. The segmentation algorithm scanned the map starting from one of its edges and proceeded line by line. Upon encountering a white pixel, the graph to which it belongs was followed using a depth-first search method [67] until the whole cluster of adjacent pixels was traversed (upper box in Fig. 1 *B* shows the segmented structure). These pixels were then excluded from further search and the procedure advanced until the whole image matrix was processed. The image processing algorithm requires no initial assumptions regarding possible distributions of the reticulum segment lengths, their clustering or extent of connectedness.

### Mathematical modeling

Image contrast adjustment, thresholding and skeletonization were done using ImageJ (US National Institutes of Health, Bethesda, MD). Segmentation analysis algorithms, statistical and visualization procedures, as well as ordinary differential equation numerical solutions were implemented in MATLAB (The MathWorks , Natick, MA). Stochastic agent-based model of the mitochondrial network was designed using Intel Corp. (Santa Clara, CA) C++ compiler and run under Linux v2.6 kernel. Random numbers were generated using VSL routines, part of Intel Corp. (Santa Clara, CA) Math Kernel Library. For non-commercial use, the computer files comprising the agent-based model can be obtained free of charge upon contacting one of the corresponding authors (the e-mail addresses are given on the first page).

## Supporting Information

Table S1 Results of segmentation analysis of confocal micrographs.
(PDF)
