# Supplementary material for: Emergence of the Mitochondrial Reticulum from Fission and Fusion Dynamics
Source: PLoS Comput Biol. 2012 Oct 25;8(10):e1002745. doi: 10.1371/journal.pcbi.1002745 (PMC3486901; doi:10.1371/journal.pcbi.1002745)
Supplement: Table S1 — Results of segmentation analysis of confocal micrographs. (PDF) [file pcbi.1002745.s001.pdf]

Supporting Information Table S1

Results of segmentation analysis of confocal micrographs

(For details of image acquisition and analysis see the main text: Materials and Methods and the first Section of Results).

Confocal micrographs	Number of branching nodes of degree k^*				Number of segments**		Number of clusters***
	$k = 3$	$k = 4$	$k = 5$	$k = 6$	in the largest cluster	total	
Image 1	718	180	23	4	351	580	65
Image 2	1245	256	28	2	255	1142	252
Image 3	834	163	5	1	202	746	155
Image 4	488	91	5	2	28	591	238
Image 5	258	42	1	0	70	242	54
Image 6	3622	737	26	3	1893	2698	252
Image 7	493	100	5	3	146	602	253

* Degree of a node is defined as number of edges connecting this node to the rest of the network.

** A mitochondrial *segment* is defined as one or more network edges connected only through bulk sites (nodes of degree 2).

*** A *cluster* is defined as a detached set of interconnected segments. The network of cellular mitochondria consists of a set of clusters.
